# Editorial: Motivation states and hedonic motivation for physical activity, exercise, and sport vs. sedentary behaviors

**DOI:** 10.3389/fspor.2023.1282118

**Published:** 2023-09-13

**Authors:** Matthew A. Stults-Kolehmainen, Genevieve Dunton, Daniel Boullosa, Garrett I. Ash, Alberto Filgueiras

**Affiliations:** ^1^ Division of Digestive Health, Yale New Haven Hospital, New Haven, CT, United States; ^2^ Department of Biobehavioral Sciences, Teachers College, Columbia University, New York, NY, United States; ^3^ Department of Population and Public Health Sciences, Keck School of Medicine, University of Southern California, Los Angeles, CA, United States; ^4^ Department of Psychology, University of Southern California, Los Angeles, CA, United States; ^5^ Faculty of Physical Activity and Sports Sciences, Universidad de León, León, Spain; ^6^ College of Healthcare Sciences, James Cook University, Townsville, QL, Australia; ^7^ Graduate Program in Movement Sciences, Integrated Institute of Health, Federal University of Mato Grosso do Sul, Campo Grande, Brazil; ^8^ Section of General Internal Medicine, Yale School of Medicine, New Haven, CT, United States; ^9^ Center for Pain, Research, Informatics, Medical Comorbidities and Education Center (PRIME), VA Connecticut Healthcare System, West Haven, CT, United States; ^10^ School of Natural, Social and Sport Sciences, University of Gloucestershire, Cheltenham, United Kingdom; ^11^ Department of Cognition and Human Development, Rio de Janeiro State University, Rio de Janeiro, Brazil

**Keywords:** motivation, affectively charged motivation states, physical activity, exercise, sedentarism, exercise psychology

**Editorial on the Research Topic** Motivation states and hedonic motivation for physical activity, exercise, and sport vs. sedentary behaviors

The concept of motivation states for physical activity and sedentarism emerged from ideas emanating from addiction medicine, self-control research, and exercise psychology. To start, Robinson and Berridge's theory of *incentive salience* (1), which seeks to understand urges and cravings for addictive substances, differentiates the notions of liking versus wanting. Those addicted to exercise experience cravings for movement (2), in other words, strong desires or wants, which are perhaps independent of the pleasure they receive from it. The want or desire to move and be active, however, is not limited to athletes or exercise addicts. Indeed, most humans experience these motivation states from time to time, if not regularly (3) (Stults-Kolehmainen et al.). In pondering over this point, there is a possibility that the reader may spontaneously feel an urge to move. Such a feeling might even persist for several minutes, but could also dissipate as quickly as it arrives. Regardless, it seems obvious that humans are more motivated to move, be active, and exercise at some moments compared to others. For instance, when waking up, the desire to move may be very weak, but after some additional time awake, a cup of coffee, and a pressing appointment in the next hour, a person might be “on fire” to move. Overall, it is clear that motivation to be physically active is a transient state that is regulated by a number of factors (Stults-Kolehmainen et al.). How this has been missed in exercise psychology textbooks is a mystery.

Typically, motivation for physical activity, and exercise and sport specifically, has been considered: (1) as a stable construct, similar to a trait (4–6), and (2) from a reflective perspective (e.g., goals). Indeed, some manuscripts in this Research Topic have adhered to this conceptualization (Zhao et al., Wu et al.). Certainly, there are enduring aspects to motivation, and this has been well-described by Self-Determination Theory (7), Hedonic Theory (8) and other meta-theories of behavior (Heredia-Leon et al.). Motivation predicts physical activity and exercise behavior (Zhao et al.), perhaps by influencing self-efficacy (Zhao et al.), attitudes, intentions (Heredia-Leon et al.), and one's satisfaction with the activity (Wu et al.). Recent health behavior theories are migrating to the idea of motivation as it varies by incidental affect and affective processing, as juxtaposed against goals and reflective processing, such as the Affect and Health Behavior Framework (9), Integrative Framework (10), and the Automatic Reflective Motivation Framework (11). These models specify motivation states as being infused with an affective component, the so-called *affectively-charged motivation states* (ACMS)—first described by Kavanaugh (12). Nevertheless, these theories and others (i.e., Affective Reflective Theory of PA) (13) have assigned a relatively minor role to motivation states. Others have taken the stance that motivation states are relevant only as they pertain to avoidance or dread of activity, particularly in the case of exercise (8), or they are overpowered by the need to minimize the exercise-related (14) or general activity effort (15).

Despite these setbacks, some conceptual (16) and measurement (3) advances have been made. Nevertheless, until recently, there was no unified effort to understand the phenomena of undulating wants or desires to move and be sedentary. In 2015, we submitted our first abstract on the topic to the North American Society for the Psychology of Sport and Physical Activity (NASPSPA) (17), with details about a 13-item instrument (i.e., the CRAVE—Cravings for Rest and Volitional Energy Expenditure scale) developed to measure motivation states. At this time, we labeled spontaneous instances of wanting to exercise “CRAVE moments”—times of pressing readiness and urgency to move, that can be experienced and/or felt in both the mind and body. Seeing more complexity in the effects, we added a concise model to explain how desires to move and be sedentary might relate to each other—the Wants and Aversions for Neuromuscular Tasks (WANT) model (16). In this heuristic, there may be genuine excitement to move, or even an urge or craving, as well as active dread or diswant (i.e., aversion) for movement. Stults-Kolehmainen et al. more formally provide ten provisions of the model, which also include ideas about how motivation states can vary with certain emotions, stressful events, and various situational factors.

This Research Topic constitutes the first attempt to meld diverse literatures into a line of scholarly work on this emerging Scientific Theory. Articles published in this series reported that motivation states:

1. predict affective responses during exercise (Do et al.), and are possibly linked to leisure satisfaction (Wu et al.),
2. are predicted by previous activity behaviors (Budnick et al.), including sitting and talking during a focus group period (Stults-Kolehmainen et al.),
3. are heightened in disorders like anorexia nervosa (Casper), Restless Legs Syndrome and other disorders (Stults-Kolehmainen et al.), and weakly related to state anxiety (Filgueiras et al.),
4. are possibly modulated by endogenous factors, like caloric imbalance and leptin concentration (Casper), but also exogenous stimuli, like music (Park et al.) and a host of other factors (Stults-Kolehmainen et al.),
5. predict intentions to be active and sedentary (Budnick et al.);
6. are linked to self-reported exercise behavior (Filgueiras et al.), and
7. follow a circadian pattern (Budnick et al.).

Measurement of motivation states was conducted with a variety of instruments. Do et al. asked participants to rate how they felt about their upcoming exercise by utilizing a sliding visual analogue scale with the endpoints being “Dreading it” to “Excited to do it”. Such a scale was used in a subsequent investigation (18), but another study from this laboratory queried participants with the phrase “Feel like exercising?”, with a response set of “yes”, “no”, and “sort of” (19) regarding the desire to be active. Stults-Kolehmainen et al. used the CRAVE scale (3), which they also translated into Portuguese and whittled down to single items for the want/desire to move and rest (Filgueiras et al.). Pedersen et al. used an unvalidated scale to measure perceived readiness to train (20), which included an item for daily motivation (i.e., “Rate your motivation to train today”). A limitation to be solved in further research with this last item is that it cannot distinguish between appetitive and reflective motivation.

People typically want what they like and are reinforced to do, and movement may provide some individuals a considerable source of reward (21). Many articles in this Research Topic highlighted the key role of exercise affective responses and pleasure/displeasure. Andersen et al. found that a traditional workout was perceived as less effortful, but also less preferable, to a more time efficient workout utilizing supersets, which produced more discomfort, but also a greater sense of pleasure, as assessed 15 min post-exercise. The same group (Pedersen et al.) found that most participants (22 out of 24 people) in a trial exploring the differences between a longer training session and two shorter sessions, preferred the longer session. They also found that the longer session was perceived as more pleasurable, even though it had higher perceptions of effort and discomfort. Timme et al. found that individuals have general preferences for engaging in activity (or not), but when making choices between active and sedentary activities, decisions are likely impacted to a greater degree by the specific options presented within a situation, and these choices are regulated by highly automated processes. Motivation states are likely under a high degree of automatic, unconscious control, and future studies should explore the interplay of these ideas. Furthermore, if exercise can be made more pleasurable or enjoyable, will people want it more? Such a possibility has yet to be tested.

In this Research Topic, we also expanded the concept of motivation states to incorporate classic ideas of drive—that the motivation to move may emanate not just from experiences of pleasure and positive reinforcement, but also negative reinforcement (Stults-Kolehmainen). In other words, are some motivation states partially the result of some pressing need to move, which has been unsatisfied (e.g., with prolonged sitting) and then prompted into consciousness? Ryan and Deci (7) highlighted a discussion from Ladygina-Kohts (22) about the drive for physical activity in primates that observed, “Active play with live creatures represents an essential need for the chimpanzee… That is why movement for the sake of movement is his unalterable, unquenchable desire… He can engage in it for hours, from dawn to sunset, day in and day out” (p. 134). It seems likely that such statements could also be made for many or most human children (and some adults). Ryan and Deci (7) have termed such tendencies “inherent propensities” and intrinsic motivation—motivated behaviors which do not require external rewards or prompts but are naturally engaged in with great interest. Flack et al. detailed how the drive to move may even result in an appetite for physical activity, perhaps a craving, and this can be altered by exercise training, though perhaps to a detriment (i.e., exercise compensation). Finally, it should be considered that the desire to move may be spurred by boredom and the need for cognitive stimulation (e.g., that comes from engaging muscles and/or moving one's physical body throughout the environment). Such perspectives seem to align with Paul Bloom's ideas of an affective “sweet spot” and “motivational pluralism”—notions that a person is motivated by more than attainment of pleasure, but also by aspects of intended pain and displeasure (23).

With the conclusion of this Research Topic, the Editors conclude that the idea of affectively-charged motivation states (ACMS) for physical activity, exercise, and sedentarism can move from the proof-of-concept stage to an emerging Scientific Theory. Important knowledge gaps were filled in, including information about intraday variation in motivation states, social influences, and how individuals think about motivation states in lay terms (Stults-Kolehmainen et al.). Significant progress was made in the measurement of motivation states, including scale development, validation, and cultural and linguistic adaptations (Filgueiras et al.). We also understand more about the reinforcing properties of exercise and factors that increase the pleasure of it. The articles in the Research Topic approached the concept from qualitative and quantitative perspectives, with observational and experimental data. Despite these advances, we underscore that these studies were mostly conducted in adults, and children likely have stronger urges to move based on motor development needs. Another gap in knowledge exists for athletes completing large amounts of movement during a competitive season, putting them at risk for burnout and neutralization of motivation. Moreover, there is great need to translate research findings into actionable strategies, such as just-in-time adaptive interventions (JITAI) (24), to promote physically active lifestyles for the greater health and well-being of all people. In this pursuit, it is clear that the next chapter for physical activity motivation begins with the “right now” or “in this very moment”. Meanwhile, we would like to thank all the authors and referees involved in this Research Topic for their outstanding scholarship.

## Author contributions

MS-K: Conceptualization, Writing – original draft, Writing – review and editing. GD: Writing – original draft, Writing – review and editing. DB: Writing – original draft, Writing – review and editing. GA: Writing – original draft, Writing – review and editing. AF: Writing – original draft, Writing – review and editing.

## Funding

The author(s) declare that no financial support was received for the research, authorship, and/or publication of this article.

GA was supported by American Heart Association Grant #852679 (GA, 2021–2024), and the National Institute of Diabetes, Digestive, and Kidney Diseases of the National Institutes of Health under a mentored research scientist development award (K01DK129441). DB was supported by Grant RYC2021-031098-I funded by MCIN/AEI/10.13039/501100011033, by “European Union NextGenerationEU/PRTR”, and by a productivity research grant (PQ1D) from CNPq (Brazil).
